# Structural mapping of mitochondrial co-translational import in cells

**DOI:** 10.1063/4.0001039

**Published:** 2025-10-27

**Authors:** Ya-Ting Chang, Benjamin A Barad, Juliette Hamid, Hamidreza Rahmani, Brian M Zid, Danielle A Grotjahn

**Affiliations:** 1 Scripps Research Institute, La Jolla, California, USA; 2 Oregon Health and Science University, Portland, Oregon, USA; 3 University of California- San Diego, San Diego, California, USA

## Abstract

Despite containing their own distinct genome, mitochondria rely heavily on the nucleus to encode 99% of the proteins necessary for mitochondrial function. Most of these proteins are synthesized by cytoplasmic ribosomes before targeting and import to mitochondria. However, a subset of proteins is imported co-translationally, with their synthesis and import occurring simultaneously. Although evidence of co-translational import into the mitochondria was discovered nearly five decades ago, the molecular mechanisms mediating this elusive process have remained unclear and somewhat debated. Our lab harnessed cellular cryo-electron tomography imaging and computational tools to provide a new molecular perspective to this elusive process. We show that cytoplasmic ribosomes engaged in co-translational import make multiple contacts with the mitochondrial outer membrane. We show that ribosomes primed for import exhibit a high degree of clustering on the mitochondrial surface in an arrangement that suggests the formation of polysomes. Interestingly, these ribosomes localize at sites of local constrictions of the outer and inner mitochondrial membrane, suggesting that local membrane remodeling may facilitate efficient protein import into the distinct mitochondrial compartments. Our work sets the stage for enabling future studies to identify molecular mechanisms mediating mitochondrial co-translational import.

